# Screening of Neonatal UK Dried Blood Spots Using a Duplex SMN1 Screening Assay

**DOI:** 10.3390/ijns7040069

**Published:** 2021-10-26

**Authors:** Stuart P. Adams, Emma Gravett, Natalie Kent, Susanne Kricke, Adeboye Ifederu, Mariacristina Scoto, Salma Samsuddin, Francesco Muntoni

**Affiliations:** 1 SIHMDS-Haematology, Camelia Botnar Labs, Great Ormond Street Hospital for Children, London WC1N 3JH, UK; emma.gravett@gosh.nhs.uk (E.G.); natalie.kent@gosh.nhs.uk (N.K.); susanne.kricke@gosh.nhs.uk (S.K.); 2 Infection, Immunity and Inflammation Section, UCL Great Ormond Street Institute of Child Health, London WC1N 1EH, UK; 3 Newborn Screening Unit, Camelia Botnar Labs, Great Ormond Street Hospital for Children, London WC1N 3JH, UK; adeboye.ifederu@gosh.nhs.uk; 4 Developmental Neurosciences Research & Teaching Department, Molecular Neurosciences, Dubowitz Neuromuscular Unit, UCL Great Ormond Street Institute of Child Health, London WC1N 1EH, UK; mariacristina.scoto@gosh.nhs.uk (M.S.); s.samsuddin@ucl.ac.uk (S.S.); f.muntoni@ucl.ac.uk (F.M.); 5 The Dubowitz Neuromuscular Centre, Great Ormond Street Hospital, London WC1N 1EH, UK

**Keywords:** spinal muscular atrophy, SMA, *SMN1*, newborn screening, NBS, dried blood spot

## Abstract

Spinal muscular atrophy (SMA) is an autosomal inherited neuromuscular genetic disease caused, in 95% of cases, by homozygous deletions involving the *SMN1* gene exon 7. It remains the leading cause of death in children under 2 years of age. New treatments have been developed and adopted for use in many countries, including the UK. Success of these treatments depends on early diagnosis and intervention in newborn babies, and many countries have implemented a newborn screening (NBS) or pilot NBS program to detect *SMN1* exon 7 deletions on dried blood spots. In the UK, there is no current NBS program for SMA, and no pilot studies have commenced. For consideration of adoption of NBS for a new condition, numerous criteria must be satisfied, including critical assessment of a working methodology. This study uses a commercially available real-time PCR assay to simultaneously detect two different DNA segments (*SMN1* exon 7 and control gene *RPP30*) using DNA extracted from a dried blood spot. This study was carried out in a routine clinical laboratory to determine the specificity, sensitivity, and feasibility of SMA screening in a UK NBS lab setting. Just under 5000 normal DBSs were used alongside 43 known SMA positive DBSs. Study results demonstrate that NBS for SMA using real-time PCR is feasible within the current UK NBS Laboratory infrastructure using the proposed algorithm.

## 1. Introduction

Spinal muscular atrophy (SMA) is one of the most common lethal recessive genetic conditions, with an incidence of 1:8000 births in Europe dependent on ethnicity [1,2]. In the UK in 2019, there were 712,699 live births [3] (UK Office for National Statistics), suggesting that approximately 71 babies were born that year with a type of SMA.

Until recently, the treatment for SMA was limited to supportive care, but in 2020, Nusinersen (an antisense oligonucleotide administered intrathecally) was approved for use in the UK by the National Institute for Health and Care Excellence (NICE). A second effective therapy (the adeno-associated viral vector onasemnogene abeparvovec) has been sanctioned for use in babies up to 12 months of age by NICE in 2021. 

Since early diagnosis and intervention is crucial for therapy to be effective, there has been considerable global interest in newborn screening (NBS) for SMA. Many countries have now adopted NBS for SMA nationally or have embarked on pilot studies regionally [4,5,6,7,8]. The methodology for SMA NBS has been fully validated elsewhere [9] and is already utilised by many NBS labs today. However, no SMA NBS assay has been tested in the UK setting previously, and thus no NBS program for SMA in the UK exists despite a clinical need for early diagnosis.

In order to provide robust laboratory data within the UK setting, we completed a short study to test a widely used, commercially available assay on anonymised, presumed normal blood spots and known SMA-positive blood spots. We were thus able to assess the suitability and feasibility of running SMA newborn screening in a routine clinical laboratory focusing on assay performance at detecting SMA with minimal false positives, and hands-on time required by staff.

## 2. Materials and Methods

This study was approved by the London–West London and GTAC Research Ethics Committee (19/LO/1801). A total of 4810 anonymised leftover dried blood spots (DBSs) were retrieved from the newborn screening department at Great Ormond Street Hospital, London. In addition, 43 known anonymised SMA-positive dried blood spots from diagnosed SMA patients were provided by Biogen Idec Ltd. (Maidenhead, UK) and were dispersed randomly among the plate runs. The assay was carried out using the RUO NeoMDx DNA Extraction kit (PerkinElmer Wallac Oy, Finland) following the manufacturers’ instructions. In short, DNA was extracted from 3.2 mm punches taken from the dried blood spots and was eluted in 80 µL Elution Solution. Subsequently, 3 µL of this eluted DNA was used in a quantitative real-time PCR (rqPCR) with 12 µL of the manufacturer’s rqPCR mix that included primer and probes for *SMN1* exon 7 and a control gene (*RPP30*). The rqPCR reactions were carried out on a Bio-Rad CFX PCR machine with the following amplification steps: 37 °C for 2 min and 94 °C for 5 min, followed by 50 cycles of 93 °C for 10 s, 60 °C for 30 s, and 69 °C for 40 s. Results were analysed using the Bio-Rad CFX Manager software (v3.1). Two sets of each kit control (C1, C2, and C3) were run with each plate. The C1 control contained no *SMN1* gene, whilst the C2 and C3 controls contained normal wild-type *SMN1* in an identical concentration.

## 3. Results

**Figure 1:**
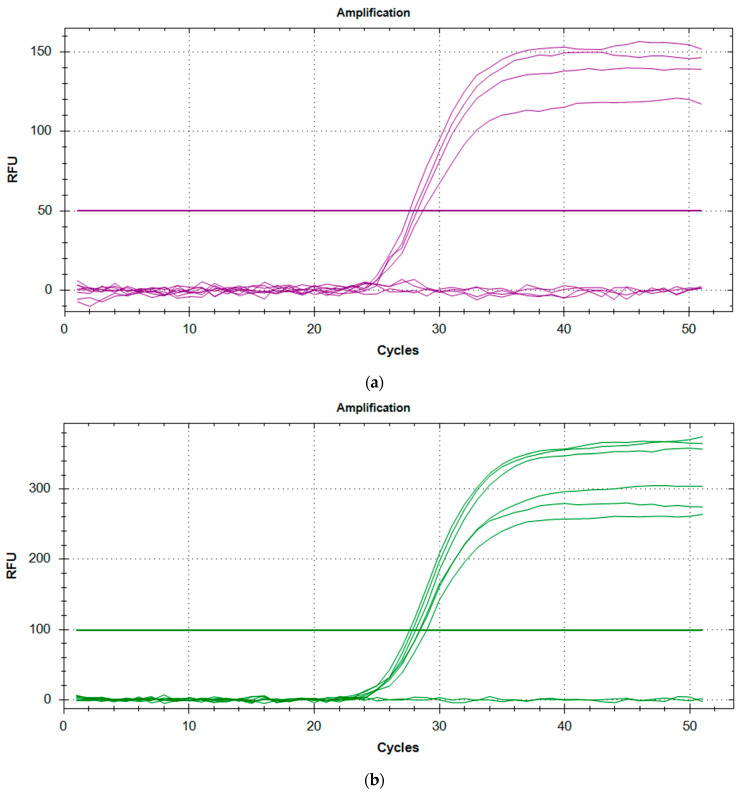
(**a**) Amplification plot for *SMN1* controls with C1 and NTC duplicates showing no amplification, and duplicates of C2 and C3 showing successful amplification. (**b**) Amplification plot for *RPP30* controls with NTC duplicates showing no amplification, while duplicates of C1, C2, and C3 show successful amplification.

After initial staff training (data from training not included in these results or analysis), all the rqPCR runs were successful with the *RPP30* controls, the *SMN1* controls, and the blank samples demonstrating consistent and reproducible amplification (Table 1, Table 2 and Figure 1). Controls and blanks were included in each run with typically one run carried out per day. This permitted reliable analysis of the anonymised, presumed normal DBS and the known SMA-positive DBS, as well as the kit controls provided in the NeoMDx PCR Reagent kit (PerkinElmer Wallac Oy). Since only one run was performed per day, the data shown in Table 1 and Table 2 illustrates interday precision for the kit controls run in duplicate. The results are within the 2 SD limits of acceptability for assay consistency and reproducibility.

**Figure 2:**
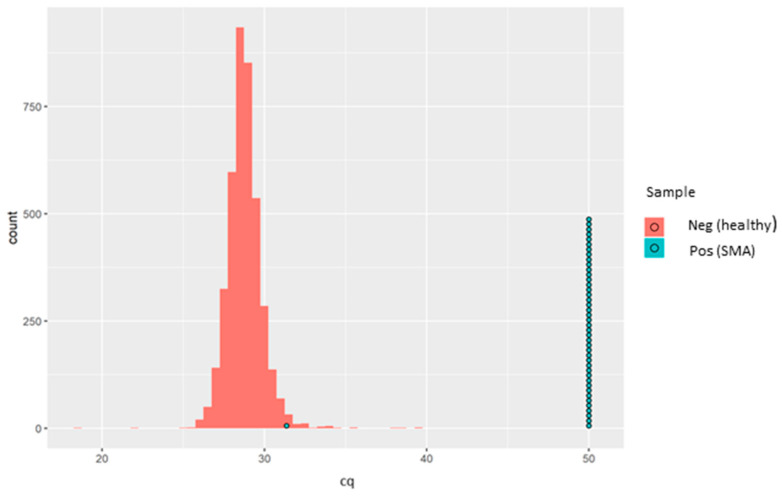
Sample box plotted in a histogram showing the Cq distribution of the healthy samples (red bars) and the SMA patients (each blue circle representing one known positive sample). One SMA-positive sample (SMA-affected sample, in turquoise) was detected as a normal healthy sample. Later, it was found that the sample was actually derived from a carrier and not from an affected individual. The aim of the kit is not to identify SMA carriers, and therefore, the results were aligned with the intended use of the kit.

Since this is qualitative (i.e., absence or presence of *SMN1* exon 7), no quantitative controls were run, and thus no calibration curve was generated. All the 4810 anonymised, presumed normal DBS samples and all 43 of the known SMA-positive samples demonstrated positive amplification for the *RPP30* control gene. Meanwhile, *SMN1* amplification was detected in all the 4810 anonymised, presumed normal DBS samples. Of the 43 known SMA-positive DBS samples, 42 showed complete absence of *SMN1* amplification. Interestingly, a single SMA-positive sample showed clear positive amplification of the *SMN1* gene with an rqPCR cycle threshold (Cq) of 31.36 (Figure 2).

It was initially presumed that this sample might have had an alternative variant in the *SMN1* gene not covered by the rqPCR assay. However, after contact with Biogen, who provided us in a blinded fashion the 43 samples with known genotypes, this “SMA positive” sample was actually derived from a carrier, and not from an affected individual, and therefore serendipitously acted as a good quality control for the assay since the aim was not to identify carriers. Thus, in our hands, this assay showed excellent analytical specificity for *SMN1* exon 7 deletion. The sensitivity of the *SMN1* amplification, based on Cq values distribution, is demonstrated in Figure 2.

## 4. Discussion

Although this assay is already widely used in numerous NBS centres, SMA NBS has never been assessed in a UK laboratory setting before. In our hands, the assay has excellent specificity and successfully identified all SMA-positive samples, and did not identify a single SMA-positive case in the presumed normal, anonymised DBS. In addition, the sample initially erroneously described as SMA positive (subsequently found to be from a carrier) showed positive *SMN1* amplification with the NeoMDx assay. This demonstrates that the assay only identifies individuals with homozygous exon 7 deletions. This is imperative for use as a screening tool for only SMA-affected individuals. 

The test was easily adopted into practice in the UK clinical laboratory setting with minimal hands-on time for staff and a simple, straightforward methodology when training new staff members. Whilst we did not investigate the results–LIMS (Laboratory Information Management System) interface capability during this study, it can be envisaged that this could easily be accommodated, given that the results can be exported via a csv file or an Excel file. The identical PerkinElmer full IVD solution (EONIS^TM^ system) comes with a preinstalled analysis software that is compatible with the LIMS systems utilised in the UK, enabling full sample tracking from punching to result interpretation. We can also now suggest that assay QC can be assessed by using the Cq of the kit controls. Any run where control Cq lies outside of the 2 SD acceptability range could be considered void.

**Figure 3:**
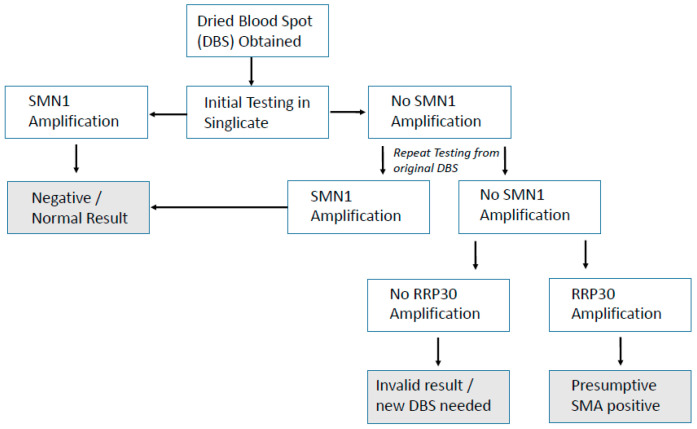
Simple, suggested algorithm for SMA NBS.

The hurdle of using DNA for NBS in the UK has already been partially overcome with the previous proof-of-concept study for SCID (severe combined immunodeficiency) NBS [10] and the recently started UK SCID NBS pilot. The method used here for SMA NBS is already being used alongside SCID NBS in many countries and can be combined as a multiplex test [9], adding very little to lab workload or cost. Hands-on time using this SMA screening assay alone was 2 h, which included punching, extracting DNA, assay setup, and analysis. Unlike SCID screening, which is a quantitative assay, SMA screening is qualitative and therefore not subject to cut-off values for result interpretation, which permitted a rapid analysis. If combined with SCID screening as a multiplex assay approach, the only extra hands-on time required would be for analysis, and this may be considered in future should SMA NBS screening be implemented in the UK. Discussion around screening for *SMN2* testing in the UK is not yet underway. Unlike for SCID screening where prematurity can be an issue affecting quantitation, this SMA assay is purely qualitative for the presence/absence of *SMN1* exon 7. An algorithm already exists in the UK for SCID NBS, and a suggested simple algorithm for SMA screening is presented in Figure 3.

In summary, our proof-of-concept study demonstrates that a simple commercially available rqPCR kit can be used in the routine clinical laboratory setting within the UK infrastructure for the detection of the exon 7 deletion in the *SMN1* gene. It is simple to use and robust and provides a direct NBS test for downstream diagnostic assessment. 

## Figures and Tables

**Table 1:** Cycle threshold (Cq) values for *RPP30* C1, C2, and C3 control samples.

*RPP30*	Minimum	Maximum	Median	Standard Deviation
C1	25.74	29.84	27.77	0.80
C2	25.68	31.69	27.83	0.82
C3	25.56	30.02	27.73	0.77

**Table 2:** Cycle threshold (Cq) values for *SMN1* control C2 and C3 samples. Control C1 is an SMA-positive (*SMN1* homozygous deletion) sample and therefore showed no amplification for *SMN1*.

*SMN1*	Minimum	Maximum	Median	Standard Deviation
C2	25.38	29.96	27.87	0.73
C3	25.22	29.44	27.70	0.76

## Data Availability

Data available on request.
